# How the R.A.M.C. Saves Combatant Wastage

**Published:** 1915-11-20

**Authors:** 


### AT A SPECIAL BASE HOSPITAL.

### HOW THE R.A.M.C. SAVES COMBATANT WASTAGE.

Whenever large armies are in the field, and especially when they are engaged in practically stationary operations in thickly populated countries, among the camp diseases which levy a great toll on the strength of the combatant forces those of the venereal group occupy no inconspicuous place, though admittedly not the chief one. It is not the least of the many services rendered by the Royal Army Medical Corps to their comrades of the fighting-line, though naturally it is not one that is frequently discussed, that the arrangements for combating this evil in our Army in Flanders are well-nigh as perfect as organisation, forethought, and modern science can make them. There have been few decorations bestowed hitherto for services rendered in base hospitals; but the D.S.O. conferred on the distinguished officer who brought into being the special venereal hospital for our troops is a notable exception, and no decoration awarded in the whole of the present campaign has been better earned or has given more general satisfaction.

A short time ago it was the writer's privilege to see the working of the hospital in question, and a most memorable impression was retained of the clockwork precision of the whole unit and the marvellous results that are obtained. Possibly this impression of the value of the treatment was enhanced by a very vivid recollection retained of an incident which occurred during the early stages of the campaign. At the very height of one of those desperate conflicts, when the thin khaki line was so nearly broken and when every man who could use rifle and bayonet was urgently needed to maintain our precarious hold, amidst the hundreds of wounded brought to an advanced dressing station within half a mile of the front trenches was a man who was suffering from nothing but a slight gleet. Three half-distracted medical officers and a handful of orderlies were doing work that might well have taxed the energies of ten times their number; but time was found for some plain-speaking to the skrimshanker, who retired to the firing-line distinctly crestfallen. In ordinary times this man would have been admitted as a patient without demur, and transferred to the base; in other words, his regiment would have lost his services just as effectively as if he had been wounded in action. It was therefore all the more encouraging to realise, at the base, how speedily cases such as this are rendered fit for active service again and returned to the firing-line.

### A Special Hospital Visited.

The hospital to which the visit about to be described was paid is one reserved for venereal diseases, for the infectious fevers, and for contagious skin complaints (scabies, impetigo, etc.). As a matter of fact at times of great stress it has perforce accommodated wounded men also—as many as 200 on a single occasion—though this has happened only rarely. In the Army at large it is not appreciated that non-venereal cases are admitted to this hospital, and the result is that a good number of patients have a sense of grievance because they feel that their admission to it is a slur upon them, or will be so regarded by their fellow-soldiers. This, however, cannot be helped; and it is possibly preferable that non-venereal cases should continue to be admitted, for thus it will in time become known that a sojourn there does not necessarily connote venereal infection.

The hospital occupies a considerable area of open meadowland on a slight slope, not very far from the seashore, but at a fair elevation above sea-level. The soil is a somewhat sticky and clayey loam, which does not lend itself well to drainage; but labour is abundant, and the earlier difficulties of keeping the camp dry have been long ago surmounted. Everything is scrupulously clean and neat, and the most apple-pie order in every section of the hospital is evidence of the splendid discipline that is preserved within it. All the buildings are temporary, and of corrugated-iron or wood construction; the accommodation is sufficient, but not luxurious, and the medical officers' quarters are no more eligible than those of the patients. Most of the fatigue work of the camp is done by the patients; the isolation (fever) section is, of course, an exception to this. There are excellent bacteriological laboratories in the camp, in which Widal and other work for the infectious fever wards is done, as well as Wassermann tests and identification of gonococci and of spirochætes. In the diagnosis of cases of syphilis, which constitute about 30 per cent. of the (venereal) admissions, great importance is attached to the finding of the spirochætes in fresh and stained films; but when the case is a clear one clinically, it is treated at once without waiting for bacteriological confirmation.

### The Treatment of Syphilis.

The total population of the hospital (patients) is about 1,500; not all of whom, be it repeated, are suffering from venereal disease, though the majority are. The admissions average about 1,300 a month; so that the average duration of stay is rather more than one month. The routine treatment of ordinary cases of syphilis is not quite as complete as it is in peace time in the Army; but it results in the restoration to duty in a state of health of so exceedingly high a percentage of the admissions that as a "business proposition" it is more economical than more elaborate and extensive courses would be. In the four weeks which is the ordinary duration of the course of treatment (for syphilis), the patient receives eight doses of arsenobenzol (Billon), which is to all intents the same as salvarsan: it is the dichlor-hydrate of dioxy-diamido-arsenobenzol, and the dosage employed is 0 3 gramme, given intravenously. During the same four weeks six doses of Lambkin's cream are also given intramuscularly, in the buttock, increasing from ½ grain to begin with up to 1 grain for the last two doses. Locally, for chancres, calomel ointment of 33 per cent. strength is used.

The room in which the arsenobenzol is administered is an object-lesson in efficient organisation. Five tables are arranged beneath a horizontal bar which is suspended from the ceiling. Each table has a (right) arm-rest; on one side of which sits a medical officer, on the other an orderly. From the horizontal bar hang two glass reservoirs, one kept full of arsenobenzol solution, the other of normal saline, each at a suitable temperature. Every four minutes five patients, stripped to the waist, enter the room and lie down on the table. Each man stretches out his right arm on the arm-rest, and the orderly at once fastens a tight rubber band around it a few inches above the elbow. The skin over the cephalic vein is then painted with a solution of iodine in chloroform, and the medical officer takes a needle in a pair of sterile forceps from a small steriliser which is kept constantly boiling. The needle is connected with the stem of a Y-shaped piece of tubing, one limb of which taps the arsenobenzol flask and the other that containing normal saline; the orderly releases the clip leading to the latter, and the medical officer plunges the needle with a single dexterous thrust into the vein. The rubber tourniquet is released, and a small quantity of saline is allowed to run, just to make sure that the needle is really in the vein, not outside it. Then the saline is disconnected, and 100 cc. of the arsenobenzol solution are run into the vein at such a rate that two minutes is spent over the flow. Then the arsenobenzol flask is shut off, and the saline cock reopened: 40 cc. of the latter fluid are allowed to run in, in order to wash out both needle and vein thoroughly, and the whole proceeding is over. The orderly drops the needle into the steriliser; the patients get up and leave the room, and a fresh quintet enter. The whole affair lasts exactly four minutes, so that each medical officer gives fifteen injections per hour. One officer's work consists solely of preparing the solution of arsenobenzol as it is required to replenish the reservoirs: so the solution is given absolutely fresh in every case. The exact technique of preparing the solution it might be indiscreet to indicate; but the preparation used, which is of French manufacture,* requires the addition of rather more alkali to neutralise it than does the original 606 of Ehrlich.

In the treatment of gonorrhœa there is nothing very original to record, except that the patients do most of their own urethral irrigation in a hut specially constructed for the purpose, after having been most carefully taught the correct methods. Furthermore, the very greatest care is taken to instruct them in the toilette of this procedure, with most gratifying results: not a single case of ophthalmia, we believe, has arisen in the entire clinic since its inception.

It would be of interest to know what steps are being taken to prevent venereal contagion of our troops in Flanders and Artois; but the problem is even more difficult than it is in our own country, for the obvious reason that our Army is not in a position to enact legislative measures in regard to the civil population, and that in the towns and villages behind the firing-line where the "resting" troops are billeted they are guests in a foreign country. It speaks volumes for the tact and sensibility of our officers, especially of those in high command, that so little friction with the civilian authorities has occurred both in this and in countless other matters. But undoubtedly the efforts of the Royal Army Medical Corps to reduce the incidence of venereal disease would have had more success if it had not been necessary to be so tender of French and Belgian susceptibilities. That is as much as can usefully be said on this subject.

* Poulenc Frères, 92 Rue Vieille du Temple, Paris.

